# Comprehensive Analysis of Ferroptosis-Related Markers for the Clinical and Biological Value in Gastric Cancer

**DOI:** 10.1155/2021/7007933

**Published:** 2021-10-27

**Authors:** Yanfei Shao, Hongtao Jia, Shuchun Li, Ling Huang, Batuer Aikemu, Guang Yang, Sen Zhang, Jing Sun, Minhua Zheng

**Affiliations:** ^1^Department of General Surgery, Ruijin Hospital, Shanghai Jiao Tong University School of Medicine, Shanghai, China; ^2^Shanghai Minimally Invasive Surgery Center, Ruijin Hospital, Shanghai Jiao Tong University School of Medicine, Shanghai, China; ^3^Shanghai Institute of Digestive Surgery, Ruijin Hospital, Shanghai Jiao Tong University School of Medicine, Shanghai, China

## Abstract

Gastric cancer is a highly malignant tumor with poor survival rate. Ferroptosis, a newly defined regulated cell death, is closely related to several tumors. Introduction of ferroptosis is promising for cancer treatments. However, the predictive role of ferroptosis in GC remains elusive. In this study, we screened the ferroptosis-related genes which were differentially expressed between normal and GC tissues. Then, based on these differentially expressed genes (DEGs), the least absolute shrinkage and selection operator (LASSO) and multivariate Cox regressions were applied to construct the 10-gene prognostic signature (*SP1*, *MYB*, *ALDH3A2*, *KEAP1*, *AIFM2*, *ITGB4*, *TGFBR1*, *MAP1LC3B*, *NOX4*, and *ZFP36*) in TCGA training dataset. Based on the median risk score, all GC patients in TCGA training dataset and GSE84437 testing dataset were classified into a high- or low-risk group. GC patients in the low-risk group showed significantly higher survival possibilities than those in the high-risk group (*P* < 0.001). Combined with the clinical characteristics, the risk score was proven as an independent factor for predicting the OS of GC patients. Besides, the GC patients in the high- or low-risk group showed significantly different GO and KEGG functional enrichments, somatic mutation, fractions of immune cells, and immunotherapy response. Then, the expression levels of these genes in signature were further verified in the GC cell lines and our own GC samples (30-paired tumor/normal tissues). Furthermore, the effects of ferroptosis inducer Erastin on these 10 ferroptosis-related genes in GC cell lines were also explored in our study. In conclusion, our study constructed a prognostic signature of 10 ferroptosis-related genes, which could well predict the prognosis and immunotherapy for GC patients.

## 1. Introduction

Gastric cancer (GC) is one of the most common malignant tumors in the world, ranking fifth for incidence (1,089,103 cases) and fourth for mortality (768,793 cases) globally in 2020 [1]. Besides, the incidence rate of GC is the highest in digestive malignant tumors in China [2]. Due to the comprehensive treatments in the last few decades, including curative surgery, chemoradiotherapy, targeted therapy, and immunotherapy, the prognosis of GC patients has improved a lot. However, most patients are diagnosed at advanced stages; the overall survival (OS) rate of 5 years remains less than 40% [3]. Currently, the prognosis of GC patients was based on the TNM staging system; nevertheless, the patients at the same stage could show obviously different prognosis. Therefore, it is necessary to identify novel and reliable biomarkers to accurately predict prognosis, to find potential therapeutic targets, and finally to improve the outcomes of GC patients.

Distinct from apoptosis, ferroptosis is a newly defined form of programmed cell death characterized by iron-dependent peroxide lipid accumulation, inducing reactive oxygen species (ROS) production and subsequent cell death [4]. Emerging evidence has demonstrated that ferroptosis plays a critical role in the redox status, cell metabolism, and multiple diseases, such as ischemia-reperfusion injury, neurodegenerative and neuropsychiatric diseases, and diverse kidney diseases [4–6]. More importantly, ferroptosis dysfunction has been implicated in the process of various tumors, including glioma [7], lung cancer [8], breast cancer [9], renal cell carcinoma [10], colorectal cancer [11], and gastric cancer [12]. Ferroptosis is inhibited in various tumors, resulting in uncontrolled proliferation of tumor cells, and is also involved in immunotherapy and drug sensitivity. Therefore, ferroptosis can serve as a promising interventional target to induce tumor cell death. Recent studies also identified some key ferroptosis-related gene signature, such as glutathione peroxidase 4 (*GPX4*), solute carrier family 7 member 11 (*SLC7A11*), nuclear respiratory factor 2 (*NRF2*), and cysteine dioxygenase type 1 (*CDO1*), which are closely related to cancer progression and patients' prognosis [13–16]. However, only few studies to date focused on the role of ferroptosis in GC, and whether ferroptosis-related genes are related to patients' prognosis and clinical treatments still needs to be fully elucidated.

In this study, we constructed and validated a ferroptosis-related gene signature, which could well predict the prognosis of GC patients. We further performed pathway and functional enrichment analysis to study the underlying mechanisms. The clinical value of the risk model based on this ferroptosis-related gene signature was also explored in immune microenvironment and tumor mutation burden of GC. In addition, the effects of ferroptosis inducer Erastin on these 10 ferroptosis-related genes in GC cell lines were also explored in our study.

## 2. Materials and Methods

### 2.1. Collection of Data

The RNA sequencing (RNA-seq) data and corresponding clinical characteristics and molecular information of gastric cancer (GC) samples (normal: 32, tumor: 375) in training cohort (TCGA-STAD) were downloaded from The Cancer Genome Atlas (TCGA) database by the “TCGAbiolinks” R package in February 2021. Similarly, the RNA-seq data of 174 normal human stomach samples in the Genotype-Tissue Expression (GTEx) database was downloaded from the University of California Santa Cruz (UCSC, https://xenabrowser.net/datapages/). Besides, the gene expression data and corresponding clinical information of the external validation cohort (GSE84437, *n* = 433; GSE29272, *n* = 268; normal: 134, tumor: 134) were downloaded from the Gene Expression Omnibus (GEO) database (https://www.ncbi.nlm.nih.gov/). Furthermore, the somatic mutation data of the TCGA-STAD was downloaded from the websites (https://portal.gdc.cancer.gov/). The 261 ferroptosis-related genes were downloaded from the FerrDb website (http://www.zhounan.org/ferrdb/), updating on 10 March 2021 [17].

### 2.2. Screening of Candidate Gene

The RNA-seq data of TCGA and GTEx datasets was normalized into the transcripts per million (TPM) data. And the scale function in the dplyr R package was employed to further normalize the RNA-seq data (TPM normalized). Batch correction was performed using the sva R package. Then, the RNA-seq data of the 261 ferroptosis-related genes was extracted to perform subsequent difference analysis. Differentially expressed gene (DEG) analysis between the normal and tumor tissues was performed by limma R package, screening out the ferroptosis-related differentially expressed genes (FDEGs). And the results of the FDEGs were visualized by ggplot2 R package. Then, univariate Cox regression analysis was performed to screen out the overall survival- (OS-) associated FDEGs which were identified as the candidate genes for subsequent establishment of prognostic ferroptosis-related gene signature.

### 2.3. Establishment and Validation of a Prognostic Ferroptosis-Related Gene Signature

In order to minimize the risk of overfitting, the least absolute shrinkage and selection operator (LASSO) regression was utilized to establish a gene prognostic signature and further screen the 10 potential hub genes from the FDEGs by the glmnet R package. Then, the protein-protein interaction (PPI) network was conducted to reveal the interaction of proteins among the protein coding between the 10 genes by the STRING database (http://www.string-db.org/). In order to explore the connection of the transcriptional level among these 10 candidate genes, the igraph and reshape2 R packages were utilized to construct correlation network of these 10 candidate genes. The multivariate Cox regression analysis based on these 10 genes was utilized to establish the prognostic ferroptosis-related gene signature. The regression coefficients of genes and their corresponding mRNA expressions were utilized to calculate the risk scores of patients. The formula of risk score was established as follows: score = ∑(corresponding mRNA expressions × regression coefficients). The median value of risk scores was utilized as the cutoff value to divide GC patients into the high- and low-risk subgroups. To test the distribution of different groups, the principal component analysis (PCA) was performed by the Rtsne and ggplot2 R packages. The survival curves were performed to analyze the prognostic status between the high- and low-risk groups by the survminer R package. And the time-dependent receiver operating characteristic curve (ROC) was performed to evaluate the predictive value of the prognostic signature by the survival and timeROC R package. Besides, the univariate and multivariate Cox regression analysis was also performed to evaluate the independent prognostic value of the prognostic signature. Furthermore, nomograms of the training and testing groups were constructed to predict the survival probability of GC patients in 1, 2, and 3 years, and their corresponding nomogram calibration curves were also constructed based on the multivariate Cox regression analysis by the rms R package.

### 2.4. Functional Enrichment Analysis

The OmicShare tools, a free online platform for data analysis (https://www.omicshare.com/tools), was employed to perform Gene Ontology (GO) and Kyoto Encyclopedia of Genes and Genomes (KEGG) analysis with *P* < 0.05 and normalized enrichment score > 1 based on the DEGs between the high- and low-risk groups. The Gene Set Enrichment Analysis (GSEA) software (https://www.gsea-msigdb.org/gsea/login.jsp/) was also utilized to further reveal the significantly enriched pathways of these DEGs. Furthermore, the maftools R package was utilized to explore and visualize the MAF files of somatic mutation data and also calculate the tumor mutation burden (TMB) scores of patients in the training group.

### 2.5. Immunotherapy Targets and Immune Infiltration Analysis

To explore the potential relationship between the 10-gene signature and the immune cell infiltration, Tumor Immune Estimation Resource (TIMER) which is a platform for analyzing the abundance of the six immune infiltration cells (macrophages, dendritic cells, neutrophils, CD4^+^ T cells, CD8^+^ T cells, and B cells) in malignant tumors was applied to evaluate the associations between the 10 hub genes and the infiltrating immune cells by Pearson correlation analysis and Student's *t*-test. And the estimate R package was utilized to explore the relationship between the risk scores and immune cell infiltration. Besides, considering the significant roles of more immune cells in the tumor microenvironment, the abundance of 22 infiltrating immune cell types in each tumor sample was calculated by CIBERSORT. Furthermore, the expression of the target genes which have been reported to be related to the immunotherapy was compared between different risk groups.

### 2.6. Expression Verification of the 10 FDEGs in the Datasets, Cell Lines, and Gastric Cancer Specimen

The differential expression levels of the 10 FDEGs between the normal and tumor stomach tissues in the training and testing groups were compared by the Wilcoxon rank-sum test. Besides, the results of the differential expression levels of the 10 FDEGs were verified by Gene Expression Profiling Interactive Analysis (GEPIA) tools (http://gepia.cancer-pku.cn/detail.php). Furthermore, a total of 30-paired normal/tumor GC specimens were recruited from Ruijin Hospital (Shanghai, China) following the guidelines set by the Ethical Committee of Ruijin Hospital. The tumor and adjacent normal stomach tissues were fixed by 10% formalin and embedded by paraffin. The optimum sections of tissue specimens were selected and deparaffinized, and immunohistochemistry (IHC) was implemented as the following antibodies: SP1 (Abcam, ab124804), NOX4 (Abcam, ab109225), AIFM2 (Proteintech, 20886-1-AP), and TFAP2C (Proteintech, 60027-1-lg). Finally, GES1, HGC-27, and MGC-803 cell lines were also applied to verify the expression of all the 10 FDEGs using real-time PCR.

### 2.7. Gastric Cancer Cell Lines and Cell Culture

GES1, HGC-27, and MGC-803 cell lines were obtained from the American Type Culture Collection (ATCC, Manassas, VA) and stored at the Shanghai Institute of Digestive Surgery. All the three cell lines were cultured in RPMI-1640 medium (Meilunbio, China) supplemented with 10% fetal bovine serum (Sunrise, Uruguay) in a humidified atmosphere at 37°C with 5% CO_2_.

### 2.8. Cytotoxicity Assay

For cytotoxicity assay, HGC-27 and MGC-803 cells were seeded in 96-well plates at a density of 5000/well and cultured in a humidified atmosphere at 37°C with 5% CO_2_ for 12 h. The ferroptosis inducer Erastin (Selleck, USA) was dissolved in dimethyl sulfoxide (DMSO) to a total concentration of 40 mM. The working concentrations were diluted to 0, 0.75, 1.5, 3, 6, 12, 25, and 50 *μ*M, and six wells were applied for each concentration. Cell proliferation was assessed using the Cell Counting Kit-8 (CCK-8; Meilunbio, China). The optical density (OD) values were measured at the 450 nm absorbance using a microplate reader. Then, the half maximal inhibitory concentration (IC_50_) values of each cell line were calculated, and the inhibition curve was plotted by ggplot2 R package.

### 2.9. Reactive Oxygen Species (ROS) Measurement

HGC-27 and MGC-803 cells were cultured in 6-well and 12-well plates for 24 h. Firstly, different working concentrations of Erastin (5, 10, and 20 *μ*M) were added to each well and treated for another 48 h. Then, these cells were washed twice with PBS and incubated with fresh RPMI-1640 medium containing 10 *μ*M 2′,7′-dichlorofluorescin diacetate (DCF; Sigma, D6883, USA) at 37°C with 5% CO_2_ for 30 min. The cells in 12-well plates were washed twice with PBS, and then, the different ROS fluorescence intensity of these cells was compared by the fluorescence microscope. In addition, the cells in 6-well plates were also washed twice with PBS and trypsinized (Meilunbio, China). The harvested cells were resuspended in PBS at 10^6^-10^7^ cells/ml, and their ROS levels were measured using flow cytometry with emission at 515-545 nm and excitation at 488 nm.

### 2.10. RNA Isolation and Real-Time PCR

Total RNA was extracted from culture cells using RNA isolator (Vazyme, China). 1 *μ*g of total RNA was reverse transcribed into complementary DNA (cDNA) using HiScript III RT SuperMix for qPCR with gDNA wiper (Vazyme, China). Then, real-time PCR was performed using ChamQ™ Universal SYBR qPCR Master Mix (Vazyme, China). The cycler protocol was 5 min at 95°C, 40 cycles of 15 s at 95°C, 60 s at 60°C, and 5 min at 72°C [18]. All primers were synthesized by Tsingke (Beijing, China) and listed in Table 1. The mRNA expression levels of the 10 candidate genes were calculated using the 2^−*ΔΔ*Ct^ method and normalized against that of GAPDH.

### 2.11. Western Blot

Cells were washed twice with PBS and then lysed with RIPA buffer containing 1% PMSF on ice for 30 min and transferred to the centrifuge tubes for centrifugation at 12000 rpm for 20 min at 4°C. The BCA assay (Beyotime, China) was used to quantify the proteins, and the equal amounts of protein were separated by 10% SDS-PAGE, transferred onto the PVDF membranes, and incubated with appropriate antibodies (SP1: Abcam, ab124804, 1 : 1000; NOX4: Abcam, ab109225, 1 : 1000; AIFM2: Proteintech, 20886-1-AP, 1 : 1000; and TFAP2C: Proteintech, 60027-1-lg, 1 : 1000) overnight at 4°C. Then, samples were incubated with anti-horseradish peroxidase-linked IgG secondary antibody (Proteintech, SA00001-1 and SA00001-2, 1 : 5000) at room temperature for 1 h and detected using chemiluminescence detection system (Tanon, China). Immunoreactive bands were measured using the sensitive ECL kit (Meilunbio, China).

### 2.12. Statistical Analysis

All the statistical analysis was conducted by the R software (version: 3.6.3) in this article. All *P* values of statistical data were based on two-sided statistical tests. *P* < 0.05 was considered to be statistically significant.

## 3. Results

### 3.1. Identification of the FDEGs

**Figure 1:**
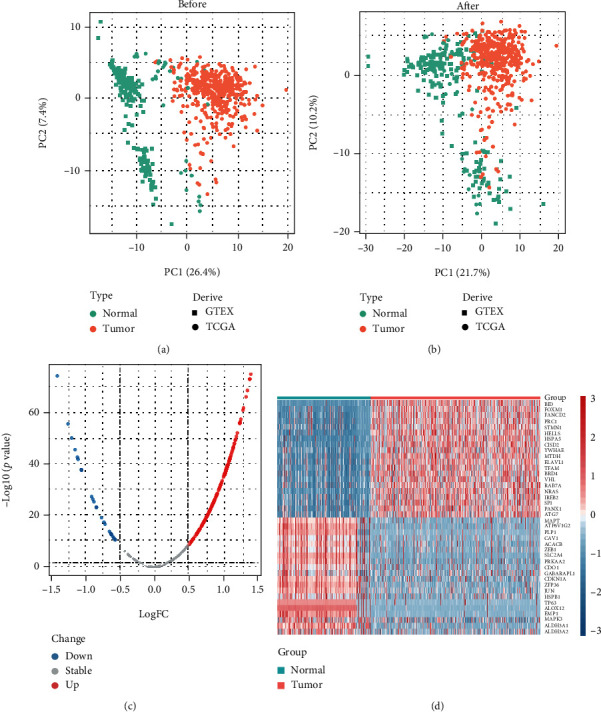
(a, b) PCA plots show batch correction between GTEx and TCGA-STAD datasets. (c) Volcano plot and (d) heat map of the FDEGs between the normal and tumor tissues from the GTEx and TCGA datasets.

Firstly, the batch effect between the GTEx and TCGA cohorts had been corrected and the results are shown in Figures 1(a) and 1(b). Then, a total of 166 ferroptosis-related genes were proven to differentially express between the 206 normal and 375 tumor stomach samples (∣log_2_FC | >0.5 and false discovery rate (FDR) < 0.01). The volcano and heat map plots were drawn to show the different expressions of ferroptosis-related genes between the normal and tumor samples based on |log_2_FC| and FDR (Figures 1(c) and 1(d)).

### 3.2. Establishment of Ferroptosis-Related Prognostic Signature

**Figure 2:**
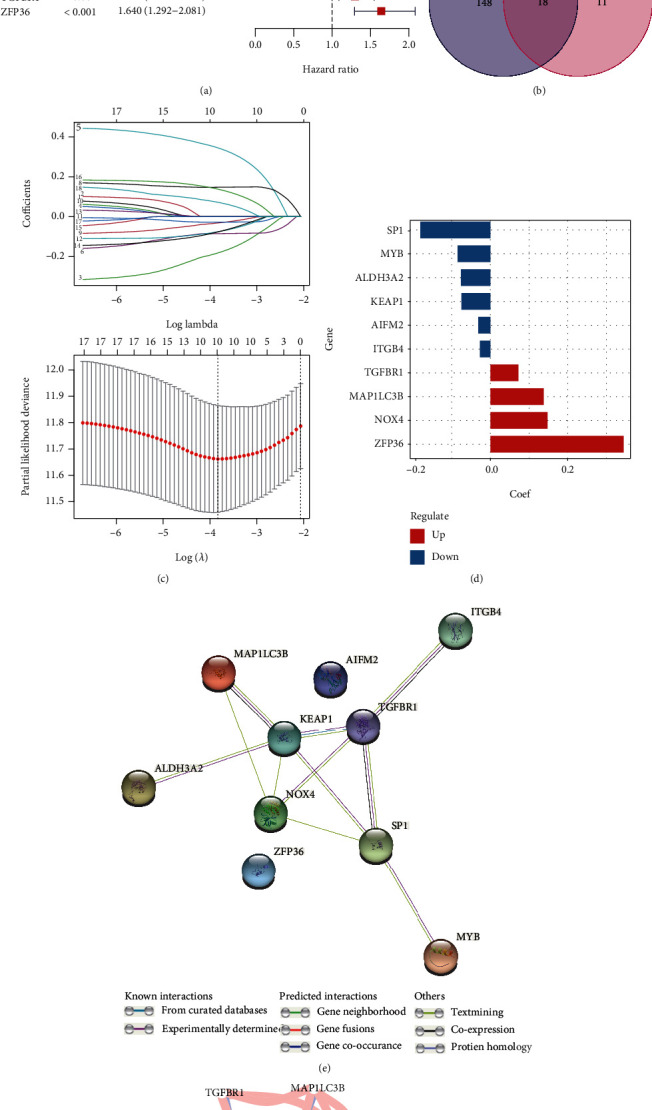
(a) Forest plot shows the results of the univariate Cox regression analysis between the expression and prognosis of ferroptosis-related genes in the training group. (b) Venn plot identifies the FDEGs that were correlated with prognosis. (c) The log lambda value and the 10 prognostic ferroptosis-related genes with nonzero coefficient. (d) Bar plot shows the coefficient of each gene. (e) PPI network constructed by STRING to indicate the interactions among these 10 genes. (f) The network plot shows the correlation among these 10 genes.

36 samples without complete OS or OS time information in TCGA training group and 136 samples whose OS time over 9 years were eliminated. Then, 29 ferroptosis-related genes were correlated with OS by the univariate Cox analysis (*P* < 0.05) in the training group and 18 of them were differentially expressed between normal and tumor tissues (Figures 2(a) and 2(b)). LASSO Cox regression analysis was utilized to construct a prognostic signature using the expression value of the 18 prognostic FDEGs mentioned above. Then, a 10-gene signature (*SP1, MYB, ALDH3A2, KEAP1, AIFM2, ITGB4, TGFBR1, MAP1LC3B, NOX4*, and *ZFP36*) was filtered out by the minimum value of lambda (*λ*) (Figure 2(c)). The coefficients of these genes are shown in Figure 2(d). And the full names, function, and coefficients of these 10 genes are shown in Table S1. According to the value of coefficients and hazard ratio (HR), the genes *TGFBR1, MAP1LC3B, NOX4,* and *ZFP36* were considered as the risk genes, while the genes *SP1, MYB, ALDH3A2, KEAP1, AIFM2,* and *ITGB4* as the protective genes. The protein interaction network among these 10 genes indicated that *NOX4*, *SP1*, and *KEAP*1 were the hub genes (Figure 2(e)). The gene correlation among them is shown in Figure 2(f). Besides, the risk scores of the signature were applied to predict prognosis in GC patients and median risk score was utilized to classify patients into the high- or low-risk groups, which was calculated as follows: risk score = (−0.181) × expression of *SP*1 + (−0.085) × expression of *MYB* + (−0.076) × expression of *ALDH*3*A*2 + (−0.075) × expression of *KEAP*1 + (−0.031) × expression of *AIFM*2 + (−0.026) × expression of *ITGB*4 + (0.072) × expression of *TGFBR*1 + (0.138) × expression of *MAP*1*LC*3*B* + (0.148) × expression of *NOX*4 + (0.345) × expression of *ZFP*36.

### 3.3. Evaluation and Validation of Ferroptosis-Related Gene Signature

**Figure 3:**
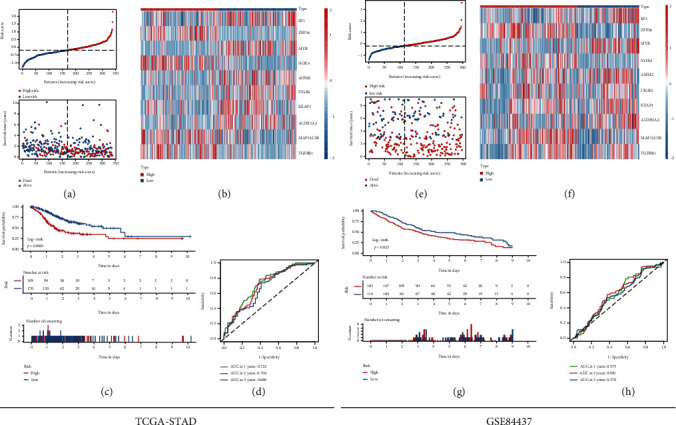
(a) The OS status and OS risk score plots of these 10 genes in the TCGA-STAD training dataset. (b) The heat map of these 10 genes between the high- and low-risk groups in the TCGA-STAD training dataset. (c) Kaplan-Meier survival curves for the OS between the high- and low-risk groups in the TCGA-STAD training dataset. (d) AUC of time-dependent ROC curve for the risk score in the TCGA-STAD training dataset. (e) The OS status and OS risk score plots of these 10 genes in the GSE84437 testing dataset. (f) The heat map of these 10 genes between the high- and low-risk groups in the GSE84437 testing dataset. (g) Kaplan-Meier survival curve for the OS between the high- and low-risk groups in the GSE84437 testing dataset. (h) AUC of time-dependent ROC curve for the risk score in the GSE84437 testing dataset.

As shown in Figure S1, PCA of the training and testing groups revealed that the patients in different risk groups could be distributed in two discrete directions. The high-risk GC patients were more likely to die earlier than low-risk patients from the results of the scatterplots (Figure 3(a)) and heat maps (Figure 3(b)). Besides, the Kaplan-Meier survival curve (Figure 3(c)) indicated that patients with low-risk scores may have a better prognosis than patients with high-risk scores in the training group. The sensitivity and specificity of the risk scores to predict prognostic features were determined from the time-dependent ROC curves by calculating the areas under the curve (AUC). And the risk scores presented the potential ability of predicting the OS status (1-year AUC = 0.722, 2-year AUC = 0.704, and 3-year AUC = 0.680) in Figure 3(d). In order to avoid the contingency of TCGA results, patients in the testing group were also classified into the low- and high-risk groups based on the median risk score. Similar to all the results above, patients with high-risk scores had a higher probability to encounter death earlier and had worse overall survival outcome than those with low-risk scores (Figures 3(e)–3(h)).

### 3.4. Analysis of Independent Prognostic Factors

**Figure 4:**
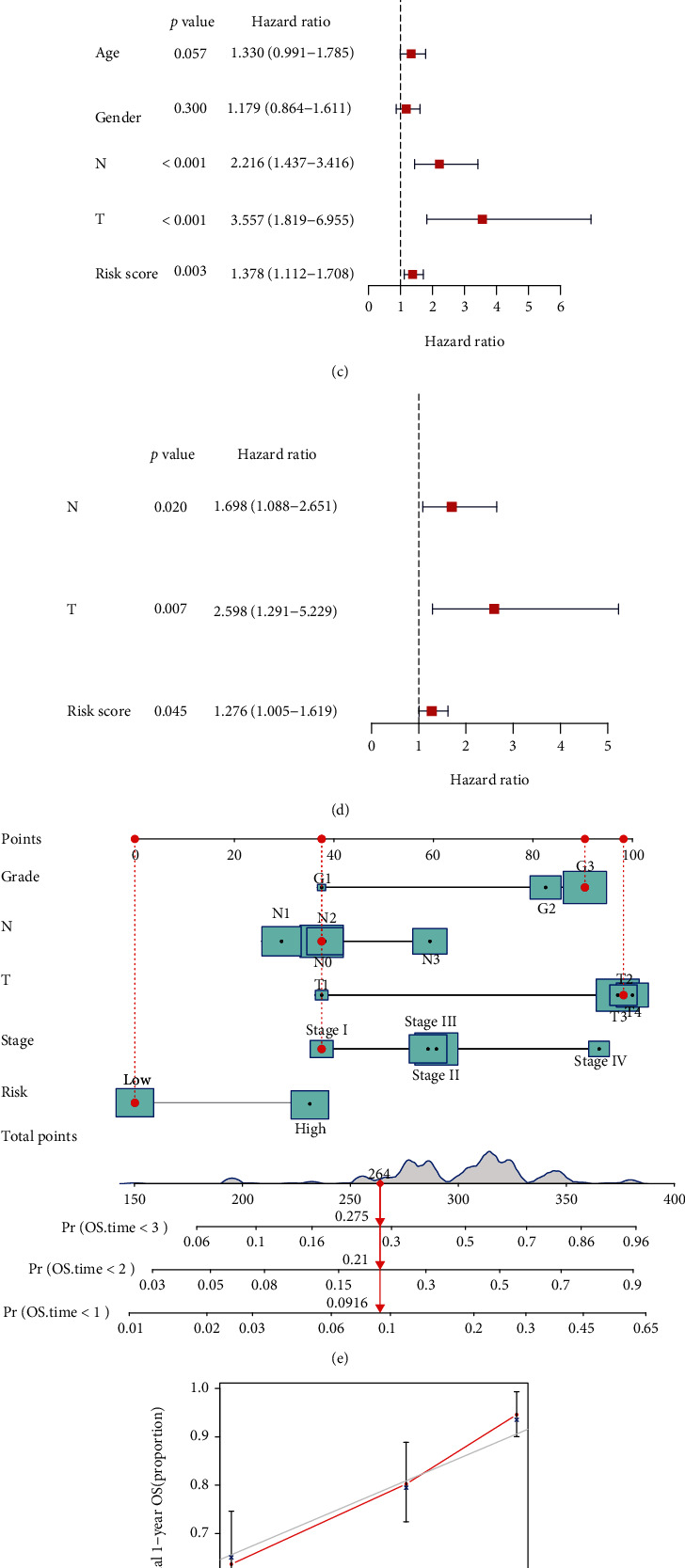
The forest plots show the results of the univariate and multivariate Cox regression analysis regarding OS in (a, b) the TCGA-STAD training and (c, d) the GSE84437 testing datasets. (e) Nomograms for predicting 3-year survival in the TCGA-STAD training dataset. (f) Calibration curves for the nomogram predicting 1- to 3-year survival in the TCGA-STAD training dataset.

The univariate and multivariate Cox regression analysis was applied to evaluate whether the risk score was the independent prognostic factor of GC patients. Firstly, in the training group, the results of the univariate Cox regression analysis showed that the risk score (*P* < 0.001, HR = 3.154, 95%CI = 2.104 − 4.728) and other clinical parameters, including T stage (*P* = 0.044, HR = 1.634, 95%CI = 1.014–2.633), N stage (*P* = 0.025, HR = 1.648, 95%CI = 1.065–2.548), and TNM stage (*P* < 0.001, HR = 1.515, 95%CI = 1.207–1.902), were significantly associated with OS (Figure 4(a)). Then, the multivariate Cox regression analysis indicated that the risk score (*P* < 0.001, HR = 3.626, 95%CI = 2.362–5.566) and TNM stage (*P* = 0.005, HR = 1.622, 95%CI = 1.155–2.277) were the independent prognostic factors of the OS (Figure 4(b)). Similar to the results above, in the testing group, the risk score was also verified to be the independent prognostic factors of the OS (Figures 4(c) and 4(d)).

### 3.5. Construction and Validation of the Nomogram Prediction Model

In order to predict the survival probability of GC patients at 1, 2, and 3 years, the clinicopathological characteristics, including grade, N stage, T stage, TNM stage, and risk score, were applied to construct the nomogram prediction model in the training group (Figure 4(e)). The corresponding calibration curves were shown to perform the good prediction in the observations in 1-3 years (Figure 4(f)). Thus, the nomogram incorporating clinical features and the risk score was stable and accurate and may be applied in the clinical evaluation of GC patients.

### 3.6. Analysis of Functional Enrichment

**Figure 5:**
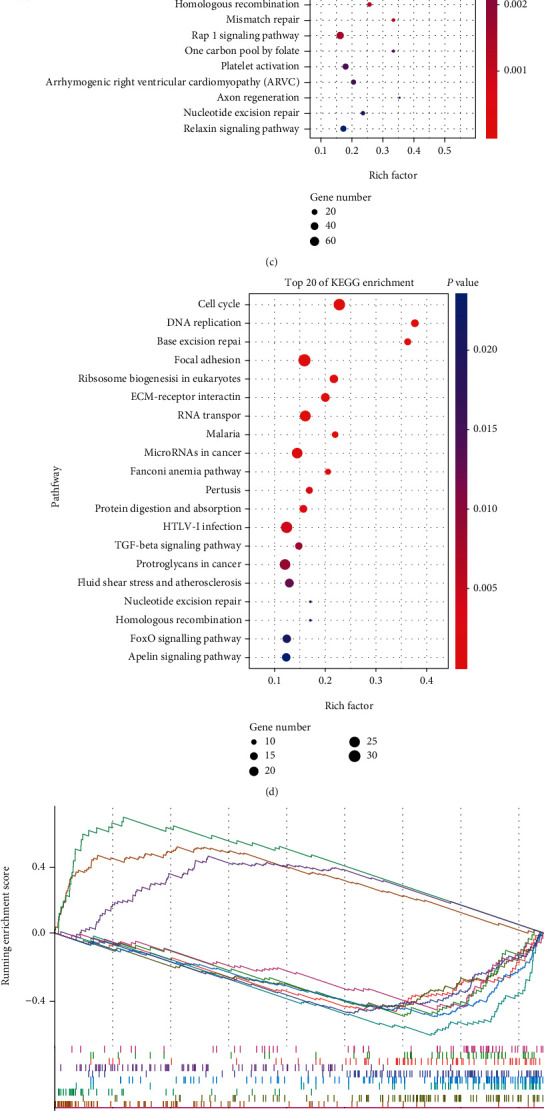
GO, KEGG, and GSEA analysis of the DEGs between the high- and low-risk groups in the (a, c, e) TCGA-STAD training and (b, d, f) GSE84437 testing datasets.

In order to further explore the signature-related downstream molecular biological functions and pathways, the GO enrichment and KEGG pathway analyses were performed by the DEGs between the high- and low-risk groups in the training and testing group. The results of GO enrichment analysis indicated that DEGs could be enriched in several tumorigenesis-related molecular functions, such as cell junction, metabolic process, immune system process, and catalytic activity in both the training and testing groups (Figures 5(a) and 5(b)). Similarly, several tumorigenesis-related pathways were also enriched based on KEGG pathway analysis, including cell cycle, regulation of actin cytoskeleton, DNA replication, ECM-receptor interaction, and focal adhesion (Figures 5(c) and 5(d)). Meanwhile, cell cycle, focal adhesion, and ECM-receptor interaction were enriched by the GSEA software and shown in Figures 5(e) and 5(f). In summary, all results of the functional enrichment analysis indicated that the risk score of the ferroptosis-related gene signature was significantly related to tumorigenesis of GC.

### 3.7. Analysis of Somatic Mutation

**Figure 6:**
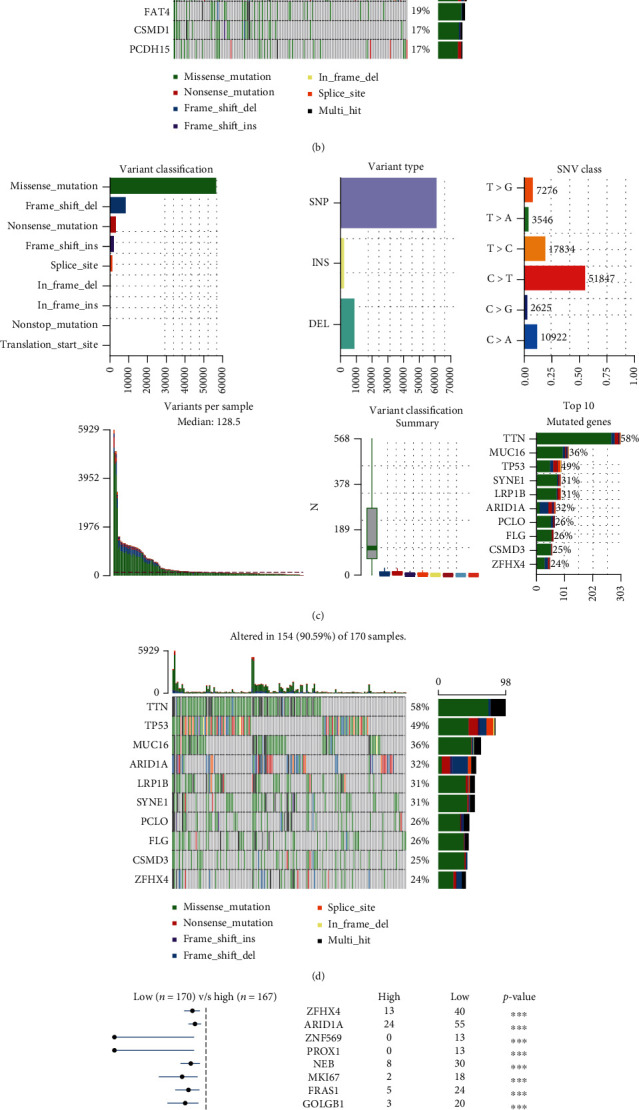
Somatic mutation summary plots and oncoplots between the (a, b) high- and (c, d) low-risk groups in the TCGA-STAD training dataset. (e) Forest plot for the differentially somatic mutation and (f) the violin plot for the TMB scores between the high- and low-risk groups in the TCGA-STAD training dataset.

Somatic mutations were closely related to the tumorigenesis of gastric cancer. To further explore the relationship between somatic mutation and risk score, simple nucleotide variation data of the high- and low-risk groups in TCGA cohort was downloaded and analyzed. The gene mutation information of the GC patients was shown in the bar and waterfall plots. Titin (*TTN*) (47%), tumor protein P53 (*TP53*) (43%), and LDL receptor-related protein 1B (*LRP1B*) (25%) were the top three genes with the highest mutation frequencies in the high-risk group and *TTN* (58%), *TP53* (49%), and mucin 16, cell surface-associated (*MUC16*) (36%) in the low-risk group (Figures 6(a) and 6(b)), while TP53 was relatively high mutated in the low-risk group (Figures 6(c) and 6(d)). The forest plot was drawn to show the difference of gene mutation distributions between the high- and low-risk groups (Figure 6(e)). Besides, tumor mutation burden (TMB) was calculated and analyzed in both groups, indicating that TMB level was significantly higher in the low-risk group (Figure 6(f)).

### 3.8. Analysis of Tumor Microenvironment and Immunotherapy Response

**Figure 7:**
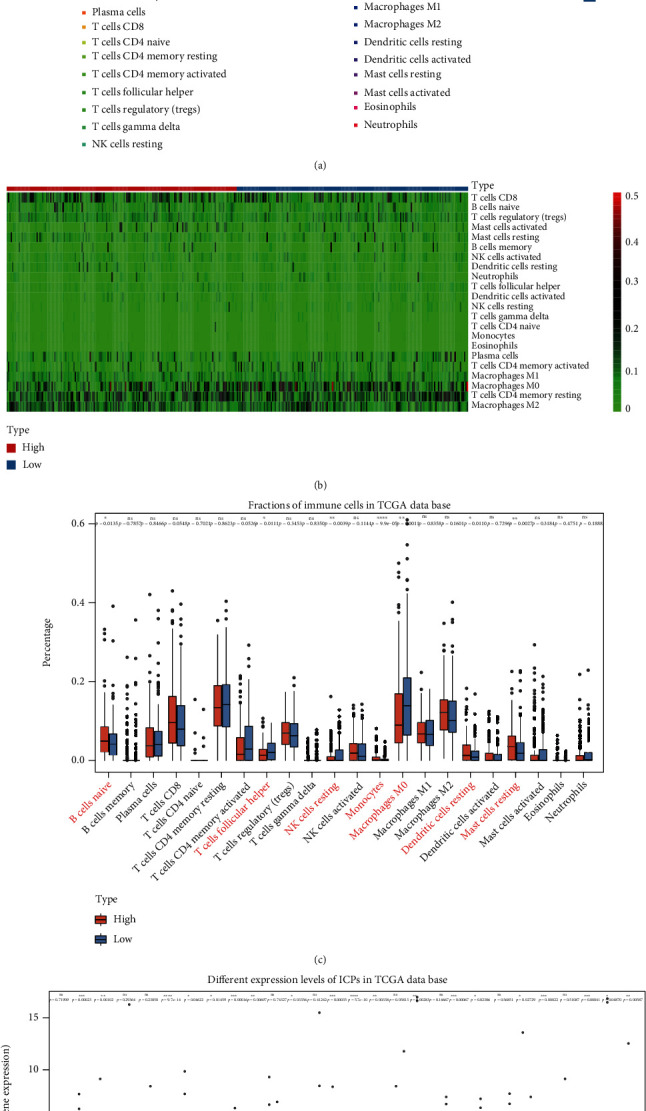
(a) Bar plot shows the proportion of 22 tumor-infiltrating immune cells (TICs) between the high- and low-risk groups in the TCGA-STAD training dataset. (b) Heat map shows the correlation between 22 TICs in the TCGA-STAD training dataset. (c) The boxplot shows the ratio differentiation of the 22 immune cells between the high- and low-risk groups in the TCGA-STAD training dataset. (d) The boxplot shows the results of the different expression levels of ICPs between the high- and low-risk groups in the TCGA-STAD training dataset. ^∗^*P* < 0.05, ^∗∗^*P* < 0.01, ^∗∗∗^*P* < 0.001, and ^∗∗∗∗^*P* < 0.0001.

According to the results of the functional enrichment analysis, immune process was significantly different between the high- and low-risk GC patients (Figure S2). Thus, TIMER and CIBERSORT analysis was utilized to further explore the relationship between risk score and tumor microenvironment. Firstly, the results of TIMER analysis indicated that the 10 FDEGs were associated with all 6 immune infiltration cells (purity, B cell, CD8^+^ T cell, CD4^+^ T cell, macrophage, neutrophil, and dendritic cell), especially for *NOX4*, *AIFM2*, and *SP1* genes (Figure S3). Meanwhile, CIBERSORT was also applied to estimate the different infiltration abundance of 22 immune cells between the high- and low-risk groups in the training group. The results showed that mast cells resting, B cells naive, dendritic cells resting, and monocytes were downregulated in the low-risk groups, while NK cells resting, macrophages M0, and T cells follicular helper were significantly upregulated (*P* < 0.05, Figures 7(a)–7(c)). Besides, the correlation analysis of risk score with common immune checkpoints (ICPs), including cytotoxic T lymphocyte-associated protein 4 (*CTLA*4), programmed cell death 1 (*PDCD1*) (PD1), *CD274* (PD-L1), hepatitis A virus cellular receptor 2 (*HAVCR2*), and lymphocyte-activating 3 (*LAG*3), was performed to estimate the immunotherapy responses through the 10-gene signature. As expected, the gene expression levels of most ICPs were significantly upregulated in the high-risk group (Figure 7(d)).

### 3.9. Validation of the Expression Levels of the 10 Ferroptosis-Related Genes

**Figure 8:**
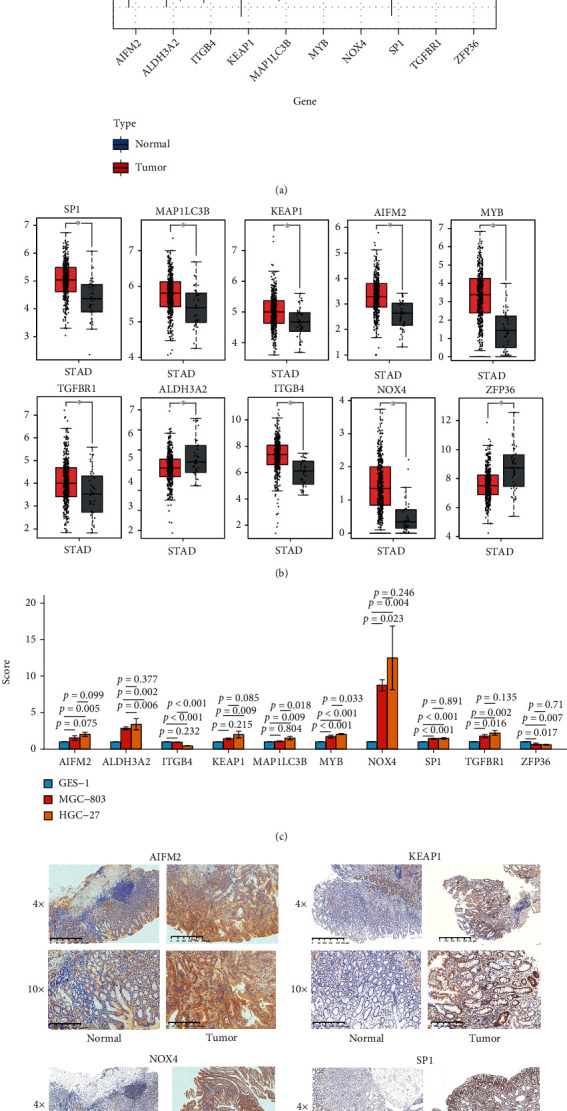
(a) The boxplot shows the expression levels of the 10 FDEGs between the normal and tumor tissues in TCGA datasets. (b) The boxplots from the GEPIA dataset verify the expression levels of the 10 FDEGs in GC patients. (c) The boxplot indicates the different expression levels of these 10 FDEGs in GC cell lines by real-time PCR. (d) Representative immunohistochemistry images of AIFM2, KEAP1, NOX4, and SP1 in GC tissues and corresponding normal tissues. ^∗^*P* < 0.05, ^∗∗^*P* < 0.01, ^∗∗∗^*P* < 0.001, and ^∗∗∗∗^*P* < 0.0001.

Compared to normal tissues, the expression levels of *SP1*, *MYB*, *KEAP1*, AIFM2, *ITGB4*, *TGFBR1*, *MAP1LC3B*, and *NOX4* were significantly upregulated, while the expression of *ALDH3A2* and *ZFP36* was downregulated in GC tumor tissues in the training group (Figure 8(a)). The same results were also verified by GEPIA (Figure 8(b)). Similarly, compared to the normal gastric cell GES1, most of these 10 genes were also differentially expressed in GC cell lines (HGC-27 and MGC-803) using real-time PCR (Figure 8(c)). Besides, we further validated the mRNA or protein expression of these 10 genes in GSE29272 and Human Protein Atlas (HPA) datasets in Figure S4. Furthermore, the expression levels of hub genes *SP1*, *KEAP1*, *AIFM2*, and *NOX4* were further verified in our GC samples. The results showed that the expression of *SP1*, *KEAP1*, *AIFM2*, and *NOX4* all increased in tumor tissues (Figure 8(d)).

### 3.10. Analysis of the Effects of Ferroptosis Inducer Erastin on the 10 Ferroptosis-Related Genes in Gastric Cancer Cell Lines

**Figure 9:**
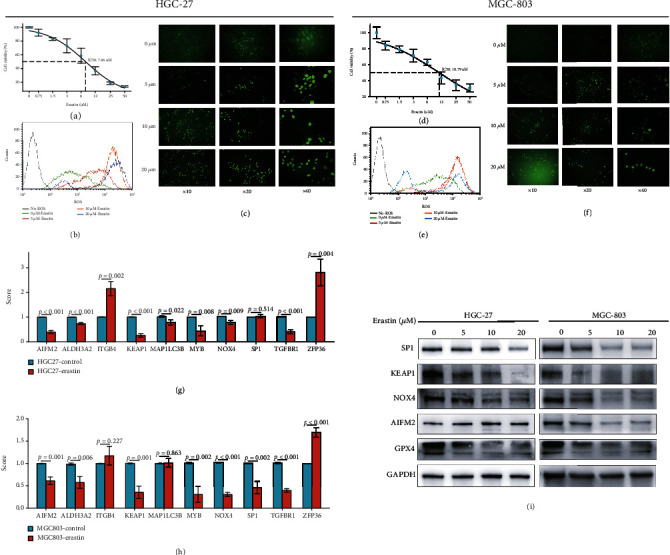
The IC_50_ curve shows the cytotoxicity assay of (a) HGC-27 and (d) MGC-803 cell lines treated with Erastin. The flow cytometry and fluorescence microscope plots verify the different ROS levels of (b, c) HGC-27 and (e, f) MGC-803 cell lines treated with Erastin. The boxplots indicate the different mRNA expression levels of these 10 FDEGs in (g) HGC-27 and (h) MGC-803 after being treated with 10 *μ*M Erastin by real-time PCR. (i) The plot detects the different protein expression levels of hub FDEGs (SP1, KEAP1, NOX4, AIFM2, and GPX4) in HGC-27 and MGC-803 after being treated with 10 *μ*M Erastin by western blot. ^∗^*P* < 0.05, ^∗∗^*P* < 0.01, ^∗∗∗^*P* < 0.001, and ^∗∗∗∗^*P* < 0.0001.

To explore the effects of the 10 ferroptosis-related genes, gastric cancer cell lines HGC-27 and MGC-803 were treated by different working concentrations of Erastin. The results of cell cytotoxicity assay indicated that Erastin could significantly inhibit the cell proliferation of HGC-27 and MGC-803 in a dose-dependent manner. The IC_50_ value of them was all around 10 *μ*M (Figures 9(a) and 9(d)). Then, according to their IC_50_, different concentrations of Erastin (5, 10, and 20 *μ*M) significantly increased the ROS both in HGC-27 and MGC-803 cell lines (Figures 9(b), 9(c), 9(e), and 9(f)). In addition, after 10 *μ*M concentration of Erastin treatment for 48 h, the mRNA expression levels of these 10 genes in HGC-27 and MGC-803 cell lines were all investigated by real-time PCR. The results indicated that the mRNA expression levels of eight genes (*AIFM2*, *ALDH3A2*, *KEAP1*, *MAP1LC3B*, *MYB*, *NOX4*, *SP1*, and *TGFBR1*) were decreased and two genes (*ITGB4* and *ZFP36*) were increased after being treated with Erastin. However, in the HGC-27 cell line, there was no statistical difference in the mRNA expression level of SP1. ITGB4 and MAP1LC3B genes were also not statistically different in MGC803-Erastin cell line (Figures 9(g) and 9(h)). In addition, similar to the mRNA expression results, the different protein expression levels of the hub genes (AIFM2, KEAP1, NOX4, and SP1) were further confirmed by western blot except AIFM2 and SP1 in the HGC-27 cell line (Figure 9(i)). In summary, the potential roles of these 10 ferroptosis-related gene markers could also be verified in cell line experiment.

## 4. Discussion

In this study, the expression level of the ferroptosis-related genes in GC tumor and normal tissues and their associations with OS were systematically investigated. A novel prognostic gene signature was established and validated in an external cohort. The independent prognostic factor, functional enrichment, somatic mutation, tumor microenvironment, and immunotherapy response analysis were performed and indicated that the ferroptosis-related gene signature can effectively predict the prognosis and clinical status for GC patients.

Ferroptosis is involved in various diseases, especially in malignant tumors [19]. Recently, several studies [12, 20–22] have proven that some ferroptosis-related genes play key roles in the process of tumorigenesis and progression of GC, but whether ferroptosis could predict the prognosis and clinical status of GC patients remains largely unknown. Usually, TNM stage system or some serum biomarkers, including CEA, CA19-9, and CA125, are used to monitor the progress and predict the prognosis of GC patients. However, these approaches are not satisfactory with low accuracy and high nonspecificity; especially, there is higher heterogeneity in GC patients. Meanwhile, with the impressive progress of bioinformatics and RNA-seq, many scholars around the world have constructed some ferroptosis-related gene signature by public databases to further explore key molecular markers and better methods to accurately predict the prognosis and drug sensitivity in several malignant tumors, including uveal melanoma, lung cancer, hepatocellular carcinoma, pancreatic cancer, and glioma [23–26]. However, few studies on gene signature had been constructed in gastric cancer.

In this study, we first screened the key ferroptosis-related DEGs in GC from the public databases. As expected, more than half of the ferroptosis-related genes were differentially expressed between adjacent nontumorous and tumor tissues in GC patients, suggesting ferroptosis plays a significant role in GC. Then, 29 of them were proven related to OS by the univariate Cox analysis, indicating that constructing a prognostic signature with these FDEGs is feasible and reasonable. Using the LASSO Cox analysis, the novel prognostic signature integrating 10 ferroptosis-related genes was identified, including *SP1*, *MYB*, *ALDH3A2*, *KEAP1*, *AIFM2*, *ITGB4*, *TGFBR1*, *MAP1LC3B*, *NOX4*, and *ZFP36*. To further explore the role of these genes in GC, we summarized their main molecular functions based on the results of this study and previous studies.

SP1, a key member of the transcription factor SP family, plays important roles in tissue development, cell differentiation, and tumor molecular biology [27]. SP1 can directly positively regulate glutathione peroxidase 4 (GPX4), which is able to significantly influence the level of lipid peroxidation and inhibit ferroptosis [28]. AIFM2 belongs to the antiferroptotic genes and is renamed as ferroptosis suppressor protein 1 (FSP1). Recent studies indicated that AIFM2 plays a significant role in ferroptosis and can act parallel to GPX4 to inhibit ferroptosis [29, 30]. ALDH3A2 is involved in preventing cellular oxidative damage by oxidizing long-chain aliphatic aldehydes. A recent study showed that ALDH3A2 can protect progenitor and leukemic stem cells from ferroptosis. Inhibiting GPX4 expression can further enhance the ferroptosis-inducing influence of ALDH3A2 depletion [31]. Different from other types of tumors, the expression of GPX4 is positively correlated with the prognosis of GC patients [12]. Thus, the potential role of SP1 and FSP1 during the process of ferroptosis in GC patients remains to be further explored. MYB has also been reported as an important transcription factor in solid tumors, which can regulate ferroportin expression, iron-related cellular activities, and tumor cell growth by modulating myeloid zinc-finger 1 [32]. Notably, a specific study indicated that MYB could inhibit Erastin-induced ferroptosis which was restrained through interacting with CDO1 in GC cells [13]. KEAP1 interacts with nuclear factor erythroid 2-related factor 2 (NRF2) in a redox-sensitive manner, and the interaction can promote the expression of gamma-glutamylcysteine synthetase [33]. In recent study, the NRF2-KEAP1 pathway is activated and upregulates SLC7A11 to inhibit ferroptosis when the expression of KEAP1 is downregulated [34]. ITGB4, a member of the integrin family, mediates cell-cell adhesion or cell growth and plays a significant role in the biology of invasive carcinoma by associating with integrin alpha 6 (ITGA6) subunit [35]. Besides, it has been reported that the induction of ferroptosis depends on cell clustering in matrix-detached cells that lack ITGB4 and ITGA6 expression [36]. TGFBR1, also known as the activin receptor-like kinase (ALK4/5), is involved in oxidative stress responses [37]. In renal proximal tubular epithelial cells, the ALK4/5 signaling pathway has been proven to be correlated with ferroptosis and blockade of the ALK4/5 signaling pathway can suppress ferroptosis [38]. Recent evidences demonstrated that autophagy facilitates ferroptosis by degrading antiferroptosis factors [39]. MAP1LC3B [40] and ZFP36 [41], key proteins of autophagy, have been considered to be correlated with ferroptosis. NOX4 is the core enzyme in mediating lipid peroxidation and promoting ferroptosis, and inhibition of NOX4 can significantly block ferroptosis [42, 43]. In summary, although these 10 genes were all correlated with ferroptosis, few studies were performed to explore their molecular functions during the process of ferroptosis in GC. Thus, to make these results more scientific, we also choose the 4 hub genes (SP1, KEAP1, AIFM2, and NOX4) of these 10 genes to further verify their expression levels in the GC cell lines and our 30-paired GC tissues by real-time PCR and immunohistochemistry. In summary, similar to the gene expression level of public databases and related studies, the expression level of them was also upregulated in GC cell lines and tissues.

Based on risk score of the 10-gene signature, GC patients can be divided into the low- and high-risk groups. Low-risk GC patients were proven to have the better prognosis and significantly longer OS than high-risk patients in both the training and testing groups. Furthermore, a series of analysis was applied to further explore the prognostic value of the signature; the results showed that the risk score was the independent prognostic factor of the OS in GC patients. Accurate nomogram prediction models can also be constructed based on the risk score. In summary, these results reveal a favorable predictive efficacy of the signature in both the training and testing groups. Meanwhile, we performed GO, KEGG, and GSEA analysis to identify the enriched biological process and pathway based on the DEGs in the high- and low-risk patients. The results showed that the cell cycle, ECM-receptor interaction, PI3K-Akt signaling pathway, and tumorigenesis-related pathways were significantly enriched both in the training and testing groups. Consistent with our results, recently, Lin et al. [44] reported that dihydroartemisinin can cause cell cycle arrest in head and neck carcinoma (HNC) cells by inducing ferroptosis. Some studies [45, 46] also demonstrated that epigenetic reprogramming of EMT promotes ferroptosis in HNC cells and gambogenic acid-induced ferroptosis inhibits the EMT in melanoma cells. Yi et al. [47] found that mutation of PI3K-Akt signaling could protect cancer cells from oxidative stress and ferroptosis.

Recent studies [48, 49] have proven that somatic mutation and tumor immune microenvironment significantly correlate with tumorigenesis, tumor progress, and drug resistance in GC patients. Wang et al. demonstrated that IFN*γ* released by CD8^+^ T cells could inhibit expression of glutamate-cystine antiporter system xc^−^, then induce tumor cell lipid peroxidation and ferroptosis, and finally improve antitumor efficacy of immunotherapy [50]. Besides, Hung et al. reported that tyrosine-protein kinase receptor TYRO3 (TYRO3) overexpression elicited anti-PD-1/PD-L1 resistance through protecting tumor cells from immunotherapy-induced ferroptosis [51]. However, the specific mechanisms of ferroptosis in tumor immunotherapy are largely unknown. In order to explore the potential mechanism of this signature, we further performed somatic mutation, tumor microenvironment, and immunotherapy response analysis. To our surprise, the somatic mutation frequency of the low-risk GC patients was higher than that of the high-risk patients and the TMB level was significantly higher in the low-risk group indicating that low-risk GC patients may be more sensitive to immunotherapy and can benefit from the immunotherapy. Based on the TMB results, we also confirmed that there were significant differences in the signature and immune checkpoints between the high- and low-risk groups. Low-risk patients had a better response to immunotherapy, suggesting that the signature has the potential to predict the immunotherapy response in GC patients. Meanwhile, the infiltration abundance of immune cells was significantly different between the low- and high-risk groups in this study. Most of the immune cells were highly infiltrated in the high-risk patients, while the abundance of NK cells was higher in the low-risk group. Previous reports indicated that increased abundance of NK cells can bring better immunotherapy efficacy [52], further suggesting the low-risk GC patients have a better response to immunotherapy. In fact, the relationship between ferroptosis and cancer immunotherapy has been reported in 2019 [50, 53], showing the sensitivity of tumor cells to ferroptosis is parallel to immune functions, which may be the reasonable explanation for the better response to immunotherapy in low-risk patients. But the exact mechanisms of how these 10 ferroptosis-related genes interact to affect tumorigenesis and immune process are still unclear and further studies are demanded.

With the gradual understanding of the role of ferroptosis in various cancers, different ferroptosis inducers have been developed as anticancer therapies, such as sulfasalazine, sorafenib, and Erastin [54, 55]. Erastin, first identified as killing tumor cells expressing oncogenic RAS, is a classical agent to induce ferroptosis by suppressing cystine/glutamate antiporter (xCT) and leading to decreased cysteine and then inhibiting the function of glutathione peroxidase 4 (GPX4) [4]. To evaluate whether these key genes play key roles in ferroptosis of GC, we applied Erastin to trigger ferroptosis in two GC cell lines, HGC-27 and MGC-803. Using CCK-8 assay, we detected significant lethal toxicity to both GC cell lines by Erastin at low concentration, showing IC_50_ value was 7.46 and 10.79 *μ*M, for HGC-27 and MGC-803, respectively. Increased ROS level is one of the features for ferroptosis. After treatment with Erastin, both cell lines showed obvious increased ROS signal by flow cytometry and fluorescence microscope, indicating Erastin-induced cell death could be attributed to ferroptosis. Besides, we also explored whether the expression of these key ferroptosis-related genes was regulated by Erastin during ferroptosis. Not surprisingly, most of the genes were dysregulated at mRNA or protein expression level, while the underlying mechanism was still not clear. Therefore, these ferroptosis-related gene could be targeted to induce cancer cell ferroptosis for future personalized therapy.

## 5. Conclusion

Ferroptosis has great potential clinical value in tumor treatments. However, the relationship between ferroptosis and tumors such as GC remains largely unclear. Thus, we systemically explored the key ferroptosis biomarkers in GC and constructed a novel ferroptosis-related gene signature which could effectively predict the prognosis of GC patients. Besides, we also further explored the signature-related downstream molecular biology functions and pathways, demonstrating the potential clinical value of this signature in somatic mutation and immunotherapy. In addition, all results had been verified by various external datasets and expression of 4 hub genes was verified in our own clinical samples. Finally, the novel prognostic signature constructed in this study needs further validation and the underlying mechanisms of ferroptosis in GC should be explored in future studies.

## Figures and Tables

**Table 1:** Real-time PCR primer sequences.

Gene	Sequence
SP1	Forward primer: TGCCTTTTCACAGGCTCGAA
	Reversed primer: TTGTGTGGCTGTGAGGTCAA
MAP1LC3B	Forward primer: TTCGAGAGCAGCATCCAACC
	Reversed primer: GATTGGTGTGGAGACGCTGA
KEAP1	Forward primer: ACGGGACAAACCGCCTTAAT
	Reversed primer: GTCCAGGAACGTGTGACCAT
AIFM2	Forward primer: TGCACCGGCATCAAGATCAA
	Reversed primer: AATGGCGTAGACGTTGCTGT
MYB	Forward primer: GATCCTGGCTCCCTACCTGA
	Reversed primer: CCAGTGGTGTGAGCAGAAGA
ALDH3A2	Forward primer: GGGATGGGAGCTTATCACGG
	Reversed primer: CACAGCGGCTACAATACCCA
ITGB4	Forward primer: TGTCCATCCCCATCATCCCT
	Reversed primer: CCCGATGGAGAGCGTAGAAC
TGFBR1	Forward primer: GTGACAGATGGGCTCTGCTT
	Reversed primer: AAGGGCCAGTAGTTGGAAGT
NOX4	Forward primer: AGCTGCCCACTTGGTGAACGC
	Reversed primer: TCAGGCCCGGAACAGTTGTGA
ZFP36	Forward primer: CCACCCCAAATACAAGACGGA
	Reversed primer: CAGGTCTTCGCTAGGGTTGT
GAPDH	Forward primer: TGAAGGTCGGAGTCAACGG
	Reversed primer: CCTGGAAGATGGTGATGGG

## Data Availability

The RNA sequencing (RNA-seq) data and corresponding clinical characteristics and molecular information of gastric cancer samples in training cohort (TCGA-STAD) were downloaded from The Cancer Genome Atlas (TCGA) database by the “TCGAbiolinks” R package in February 2021. The RNA-seq data of normal human stomach samples in GTEx database was downloaded from the University of California Santa Cruz (UCSC, https://xenabrowser.net/datapages/). Besides, the gene expression data and corresponding clinical information of the external validation cohorts (GSE84437, GSE29272) were downloaded from the GEO database (https://www.ncbi.nlm.nih.gov/). Furthermore, the somatic mutation data of the TCGA-STAD was downloaded from the websites (https://portal.gdc.cancer.gov/). The 261 ferroptosis-related genes were downloaded from the FerrDb website (http://www.zhounan.org/ferrdb/), updating on 10 March 2021.

## Supplementary Materials

Supplementary Materials Figure S1: PCA plots of the TCGA-STAD training and GSE84437 testing datasets. Figure S2: KEGG circular and pathway annotation plots of the TCGA-STAD training and GSE84437 testing datasets. Figure S3: the diagrams of the correlation analysis between these 10 FDEGs and the immune infiltration level in TCGA dataset by TIMER. Figure S4: validation of the mRNA or protein expression of these 10 genes in GSE29272 (except *AIFM2*) and HPA (except NOX4) datasets. Table S1: full names, function, and coefficients of the 10 genes.
