# Supplementary material for: Comprehensive Analysis of Ferroptosis-Related Markers for the Clinical and Biological Value in Gastric Cancer
Source: Oxid Med Cell Longev. 2021 Oct 27;2021:7007933. doi: 10.1155/2021/7007933 (PMC8566081; doi:10.1155/2021/7007933)
Supplement: Supplementary Materials — Figure S1: PCA plots of the TCGA-STAD training and GSE84437 testing datasets. Figure S2: KEGG circular and pathway annotation plots of the TCGA-STAD training and GSE84437 testing datasets. Figure S3: the diagrams of the correlation analysis between these 10 FDEGs and the immune infiltration level in TCGA dataset by TIMER. Figure S4: validation of the mRNA or protein expression of these 10 genes in GSE29272 (except AIFM2) and HPA (except NOX4) datasets. Table S1: full names, function, and coefficients of the 10 genes. [file 7007933.f1.zip › 7007933.f1/Supplementary File-Table S1.pdf]

Full names, function and coefficients of the 10 genes

Gene	Full name	Function	Coefficient
SP1	Sp1 Transcription Factor	The protein encoded by this gene is a zinc finger transcription factor that binds to GC-rich motifs of many promoters. The encoded protein is involved in many cellular processes, including cell differentiation, cell growth, apoptosis, immune responses, response to DNA damage, and chromatin remodeling.	-0.181
ZFP36	ZFP36 Ring Finger Protein	Positively regulates early adipogenesis of preadipocytes by promoting ARE-mediated mRNA decay of immediate early genes (IEGs).Plays a role in the regulation of proliferation, differentiation and apoptosis.	0.345
MYB	MYB Proto-Oncogene, Transcription Factor	This gene encodes a protein with three HTH DNA-binding domains that functions as a transcription regulator. This protein plays an essential role in the regulation of hematopoiesis.	-0.085
NOX4	NADPH Oxidase 4	This gene encodes a member of the NOX-family of enzymes that functions as the catalytic subunit the NADPH oxidase complex. The encoded protein is localized to non-phagocytic cells where it acts as an oxygen sensor and catalyzes the reduction of molecular oxygen to various reactive oxygen species (ROS).	0.148

AIFM2	Apoptosis Inducing Factor Mitochondria Associated 2	A NAD(P)H-dependent oxidoreductase involved in cellular oxidative stress response. Cooperates with GPX4 to suppress phospholipid peroxidation and ferroptosis.	-0.031
ITGB4	Integrin Subunit Beta 4	Integrins mediate cell-matrix or cell-cell adhesion, and transduced signals that regulate gene expression and cell growth. Plays a critical structural role in the hemidesmosome of epithelial cells. Is required for the regulation of keratinocyte polarity and motility.	-0.026
KEAP1	Kelch Like ECH Associated Protein 1	This gene encodes a protein containing KELCH-1 like domains, as well as a BTB/POZ domain. KEAP1 acts as a key sensor of oxidative and electrophilic stress.	-0.075
ALDH3A2	Aldehyde Dehydrogenase 3 Family Member A2	Aldehyde dehydrogenase isozymes are thought to play a major role in the detoxification of aldehydes generated by alcohol metabolism and lipid peroxidation. Catalyzes the oxidation of medium and long chain aliphatic aldehydes to fatty acids.	-0.076

MAP1LC3B	Microtubule Associated Protein 1 Light Chain 3 Beta	Plays a role in mitophagy which contributes to regulate mitochondrial quantity and quality by eliminating the mitochondria to a basal level to fulfill cellular energy requirements and preventing excess ROS production.	0.138
TGFBR1	Transforming Growth Factor Beta Receptor 1	Transduces the TGFB1, TGFB2 and TGFB3 signal from the cell surface to the cytoplasm and is thus regulating a plethora of physiological and pathological processes including cell cycle arrest in epithelial and hematopoietic cells, control of mesenchymal cell proliferation and differentiation, wound healing, extracellular matrix production, immunosuppression and carcinogenesis.	0.072
