# Supplementary figures and images for: Comprehensive Analysis of Ferroptosis-Related Markers for the Clinical and Biological Value in Gastric Cancer
Source: Oxid Med Cell Longev. 2021 Oct 27;2021:7007933. doi: 10.1155/2021/7007933 (PMC8566081; doi:10.1155/2021/7007933)

# Figure S1

A

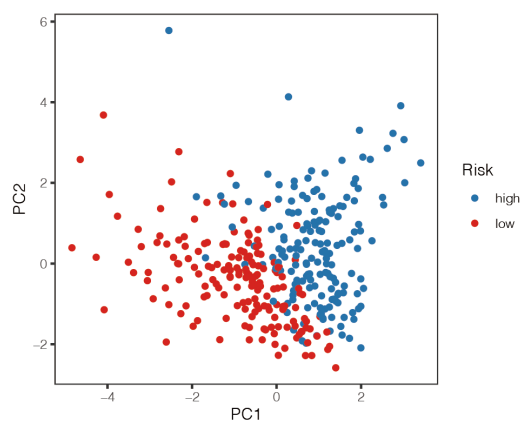

B

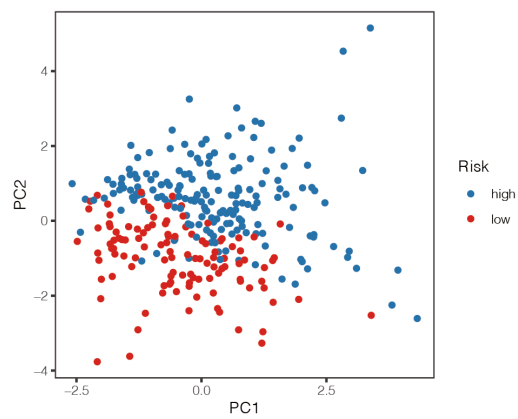

# Figure S2

TCGA-STAD

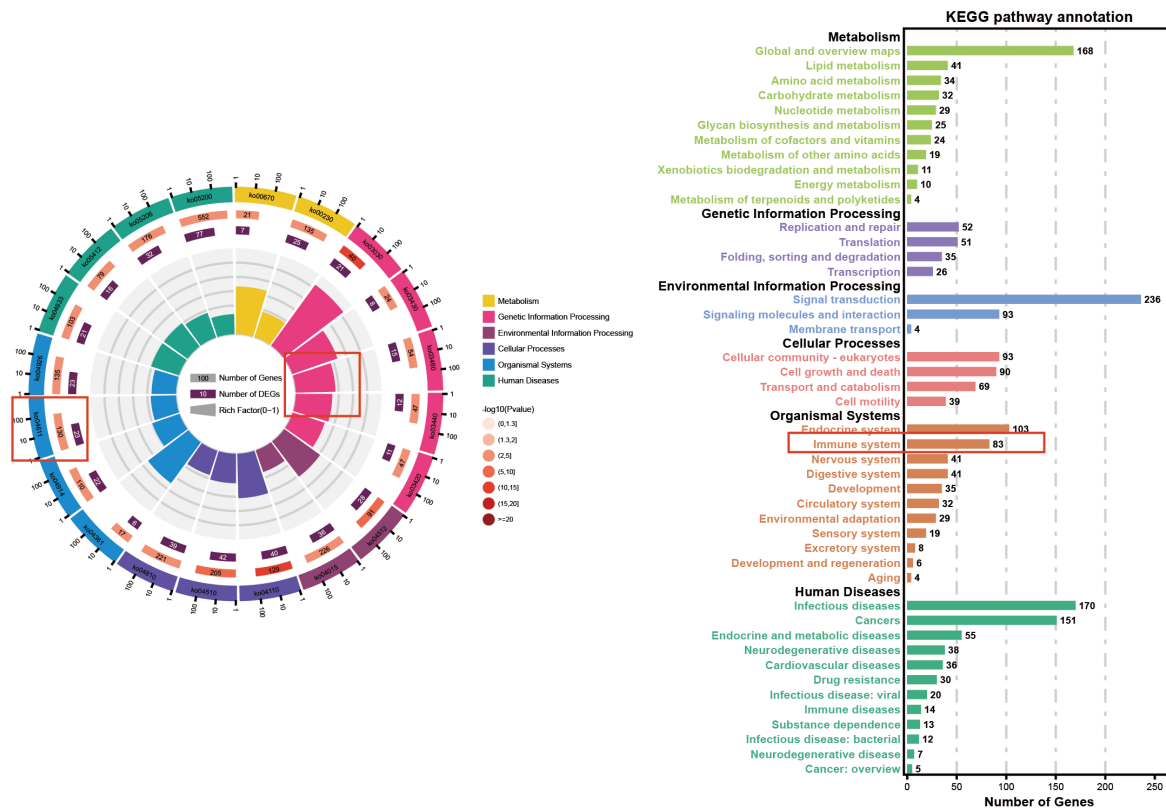

GSE84437

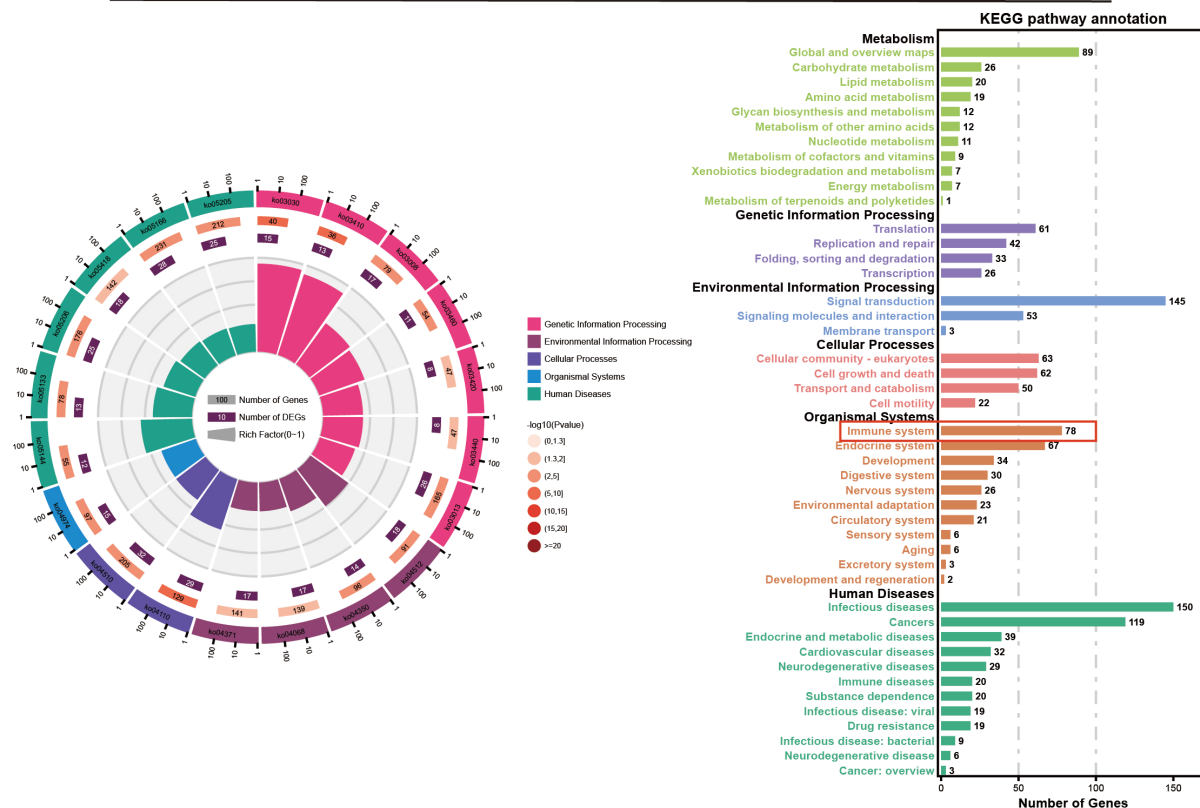

# Figure S3

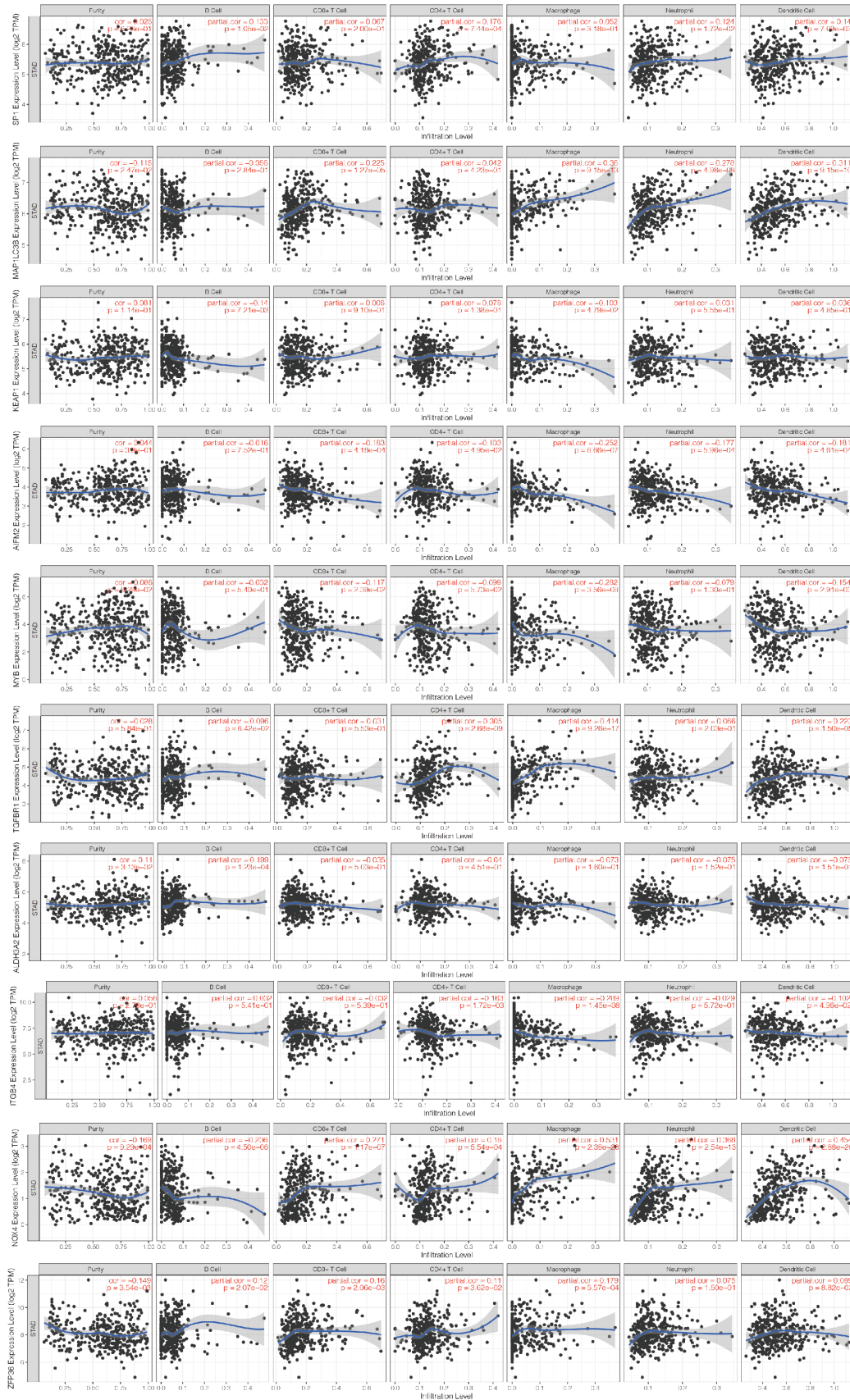

Figure S4

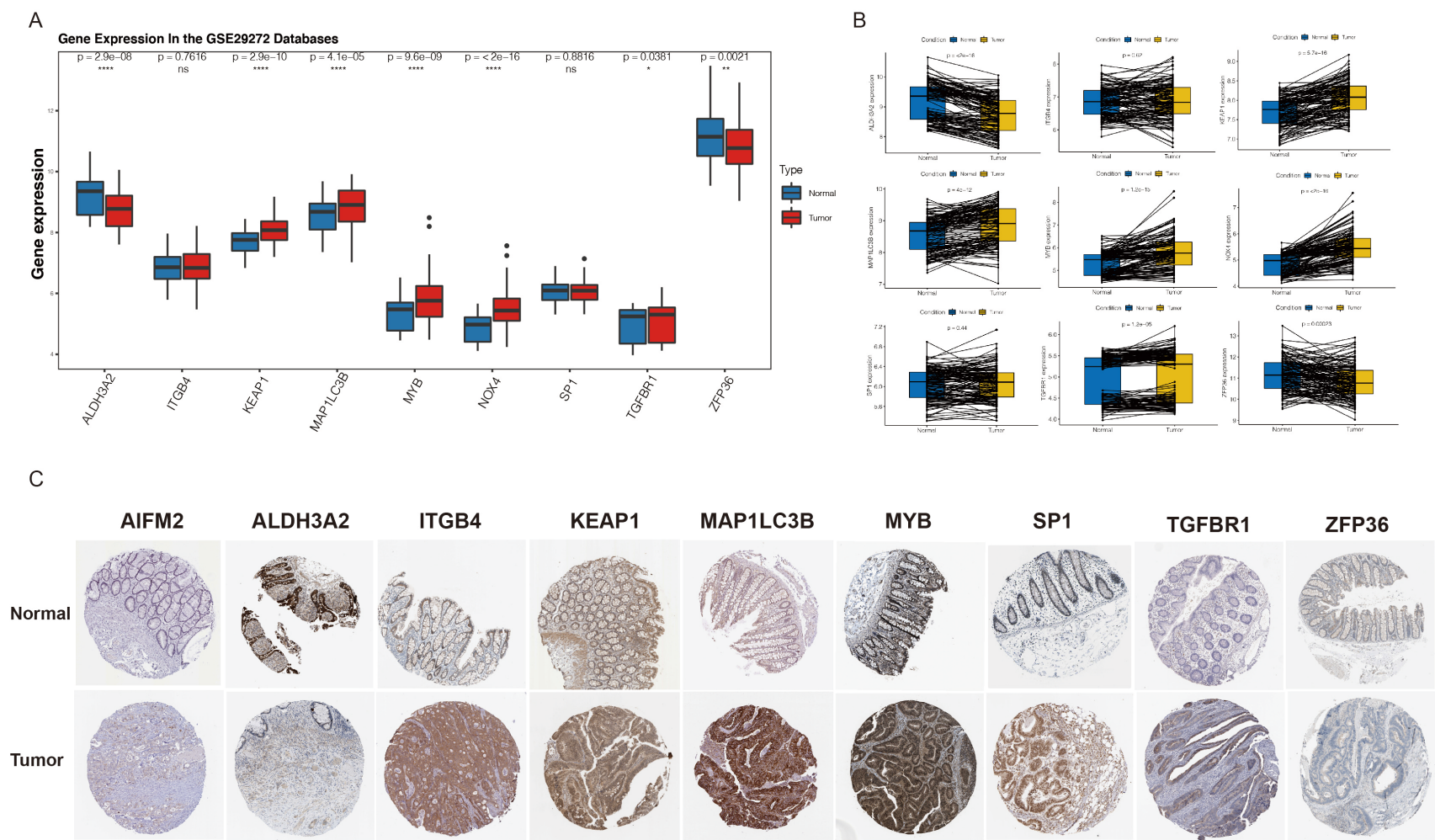

Supplement: Supplementary Materials — Figure S1: PCA plots of the TCGA-STAD training and GSE84437 testing datasets. Figure S2: KEGG circular and pathway annotation plots of the TCGA-STAD training and GSE84437 testing datasets. Figure S3: the diagrams of the correlation analysis between these 10 FDEGs and the immune infiltration level in TCGA dataset by TIMER. Figure S4: validation of the mRNA or protein expression of these 10 genes in GSE29272 (except AIFM2) and HPA (except NOX4) datasets. Table S1: full names, function, and coefficients of the 10 genes. [file 7007933.f1.zip › 7007933.f1/Supplementary File-Figure R1.pdf]
